# Health-Related Outcomes among the Poor: Medicaid Expansion vs. Non-Expansion States

**DOI:** 10.1371/journal.pone.0144429

**Published:** 2015-12-31

**Authors:** Xuesong Han, Binh T. Nguyen, Jeffrey Drope, Ahmedin Jemal

**Affiliations:** 1 Intramural Research, American Cancer Society, Atlanta, Georgia, United States of America; 2 Rollins School of Public Health, Emory University, Atlanta, Georgia, United States of America; University of Pennsylvania, UNITED STATES

## Abstract

**Introduction:**

States’ decisions not to expand Medicaid under the Affordable Care Act (ACA) could potentially affect access to care and health status among their low-income residents.

**Methods:**

The 2010–2012 nationally representative Medical Expenditure Panel Survey data were analyzed in 2015 to compare 9755 low-income adults aged 18–64 years from Medicaid-expanding states with 7455 adults from nonexpanding states. Multivariate logistic regression models were fitted to evaluate the differences in access to care, receipt of preventive services, quality of care, attitudes about health and self-reported health status by Medicaid expansion status. The differences in care utilization and medical expenditures between the two groups were examined using a 2-part modeling approach.

**Results:**

Compared to their counterparts in Medicaid expansion states, low income adults in the nonexpanding states were more likely to be black and reside in rural areas and were less likely to have a usual source of care (prevalence ratio[PR] 0.86, 95% confidence interval[CI] 0.82–0.91) and recommended preventive services such as dental checkups (PR = 0.86; CI = 0.79–0.94), routine checks (PR = 0.89; CI = 0.83–0.95), flu vaccinations (PR = 0.89; CI = 0.81–0.98), and blood pressure checks (PR = 0.96; CI = 0.94–0.99). They also had less care utilization, fewer prescriptions, and less medical expenditures, but more out-of-pocket expenditures (all p-value <0.05).

**Conclusions:**

Low-income adults in Medicaid nonexpanding states, who are disproportionately represented by blacks and rural residents, were worse off for multiple health-related outcomes compared to their counterparts in Medicaid expanding states at the baseline of ACA implementation, suggesting that low income adults residing in nonexpanding states may benefit markedly from the expansion of Medicaid.

## Introduction

The Affordable Care Act (ACA) was designed to expand Medicaid coverage to all U.S. residents with family income at or below 138% of the federal poverty level (FPL), including non-disabled childless adults who were previously not eligible for Medicaid in most states [1]. In 2012, a US Supreme court ruling affirmed states’ rights to decide whether to expand Medicaid eligibility as promulgated by the ACA [2]. As of November, 2015, 31 states including the District of Columbia have adopted Medicaid expansion under the ACA, while 19 states have opted out and 1 states continue to discuss expansion [3]. States’ policy choices regarding expansion will likely affect the health options of the low-income population and potentially lead to disparities in access to health care and health status between the two sets of states. For example, a recent examination of the nation’s four largest states, New York and California who expanded Medicaid eligibility, and Florida and Texas who declined expansion found that rates of access to care problems due to cost and medical bill problems were lower in New York and California [4]. In this study, we systematically examined whether there are differences in health care need, utilization and financial burden between low income populations residing in Medicaid expansion states and nonexpanding states at baseline using a national interview survey.

## Materials and Methods

### Study sample

The study sample was based on pooled data from 3 years (2010–2012) of the Medical Expenditure Panel Survey (MEPS) Household Component, which is a nationally representative survey of the US civilian non-institutionalized population conducted by the Agency for Healthcare Research and Quality (AHRQ). The MEPS collects data on health insurance, and health care access, utilization, and expenditures through household interview, supplemented by a self-administered questionnaire (SAQ) on health and health opinions. More information about the survey design and contents can be found online [5]. The combined average annual response rate for 2010–2012 was approximately 55%. Verbal consent was obtained from household respondent and documented by the fact that the interview was conducted. Informed consent procedure included a video and written materials mailed to the household. Written consent was not obtained due to logistic and administrative difficulties. MEPS and its consent procedure have been reviewed and approved by the Westat Institutional Review Board.

Adults aged 18–64 years with a family income ≤ 138% FPL (N = 17212) were identified, including 9755 from the Medicaid expanding states (AZ, AR, CA, CO, CT, DE, DC, HI, IL, IN, IA, KY, MD, MA, MI, MN, NV, NH, NJ, NM, NY, ND, OH, OR, PA, RI, VT, WA, WV) and 7457 from the nonexpanding states (AL, FL, GA, ID, KS, LA, ME, MS, NE, NC, OK, SC, SD, TX, WI, AK, MO, MT, TN, UT, VA, WY), respectively representing 19.5 million and 22.3 million residents in the nation. The categorization reflects states’ decision on Medicaid expansion in February 2015 when we performed the analysis. The S1 Appendix provides population size, poverty rate and Medicaid status for each state. We did not limit our analysis to only uninsured low-income population because not only the uninsured, but also the underinsured (e.g. those with high deductible private insurance plans) would all potentially benefit from Medicaid expansion.

### Measures

Demographic characteristics used in the analysis include gender, age, race/ethnicity, marital status, education, employment status, residence in a metropolitan statistical area (MSA) and health insurance. Chronic conditions were ascertained with a series of questions about whether a person was ever diagnosed with any of the following conditions: high blood pressure, stroke, heart disease, emphysema, high cholesterol, cancer, diabetes, arthritis and asthma. The number of conditions was enumerated for each person and categorized as 0, 1 and “2 or more.” Body mass index (BMI) was calculated based on reported height and weight and categorized as underweight (BMI<18.5 kg/m^2^), normal weight (18.5 to <25 kg/m^2^), overweight (25 to <30 kg/m^2^) or obese (≥30 kg/m^2^). Current smoking status was included.

Health-related outcomes included:

**Perceived access to care** was measured by a series of questions: if the individual has a usual source of health care; whether the person was unable to get or delayed in receiving treatment of medical care, dental care or prescription medicine.

**Realized access to preventive care** was measured by questions about preventive services use and cancer screening recommended by health professional guidelines [6, 7]. Preventive services included a dental checkup at least once a year, a routine physical checkup within the past year, a flu vaccination within the past year, a blood pressure check within the past 2 years, and a cholesterol check within the past 2 years. Cancer screening was assessed among age- and gender-eligible women and men: a Pap test within the past 3 years in women aged ≥ 21 years without a hysterectomy or a history of cervical cancer, a mammogram within the past 2 years in women aged 40 or older without a history of breast cancer, and colorectal cancer screening among adults aged ≥ 50 years without a history of colorectal cancer, including a blood stool test within the past year, a sigmoidoscopy within the past 5 years or a colonoscopy within the past 10 years.

**Care utilization and expenditures** information was available annually for each participant by service type (number of office-based visits, outpatient visits, emergency room visits, inpatient night stays, dental visits and prescriptions). Expenditures were also available by payment source (out of pocket; private insurance/TRICARE; government including Medicare, Medicaid, Veteran’s Administration/CHAMPVA and Workers’ Compensation; or other).

**Quality of care** was measured with SAQ from the health plan version of CAHPS^®^ (Consumer Assessment of Healthcare Providers and Systems), an AHRQ-sponsored family of survey instruments designed to measure quality of care from the consumer’s perspective. We examined the frequency of “always” to the following three questions during the past 12 months: 1) if had an illness, injury or condition needing care right away, how often received the care right away; 2) if any appointment was made with a doctor or clinic for health care, how often an appointment was made as soon as he/she thought it was needed; and 3) if visited doctor and any care, tests or treatment was believed necessary, how often it was easy to access the care.

**Attitudes about health** was assessed in SAQ with two items for attitudes toward health insurance and another two items for attitudes that might influence decisions to purchase health insurance or to use health services: 1) do not need health insurance; 2) health insurance is not worth the money it costs; 3) more likely to take risks than the average person; and 4) can overcome illness without help from a medically trained person. For each item, one of the five options was selected: disagree strongly, disagree somewhat, uncertain, agree somewhat, and agree strongly. We dichotomized our outcomes as “disagree strongly” or not.

**Health status** was generated via multiple questions. Perceived health status and perceived mental health status were rated as excellent, very good, good, fair or poor at the household interview. The SAQ contained three measures of health status: 1) the Short-Form 12 Version 2 (SF-12v2) [8], from which scores of the Physical Component Summary (PCS) and the Mental Component Summary (MCS) were calculated and dichotomized based on the mean of the age- and gender- specific norms [9]; 2) Kessler -6 (K6) scale for non-specific psychological distress [10], where a score of ≥ 13 indicating serious psychological distress [11]; and 3) Patient Health Questionnaire (PHQ-2) [12], where a score of ≥ 3 indicating depressive symptoms in screening [13].

### Statistical analysis

The access-restricted de-identified data file was used to acquire the resident state of the participants, and the participants were assigned to one of two groups based on their states’ Medicaid expansion status (Yes or No/under discussion). Demographic characteristics were described for expansion and non-expansion groups separately, and their difference was tested using Wald Chi-square tests. The crude rate and 95% confidence interval (CI) were calculated for each health-related outcome in the expansion and non-expansion groups. Multivariate logistic models were fitted to calculate the prevalence ratio (PR) and 95% CI [14] for occurrence of each health-related outcome associated with Medicaid expansion status controlling for sex, age, race/ethnicity, marital status, education, MSA residence and number of chronic conditions, using the expansion group as the reference. For care utilization and medical expenditures, the crude mean and standard deviation were calculated. Adjusted difference between the expansion and non-expansion groups and 95% CI were calculated using a 2-part modeling approach with a logistic regression in part 1 and a Poisson regression (for health care utilization) or generalized gamma regression with log link (for medical expenditures) in part 2 to account for the zero values in the utilization and expenditure data [15, 16], controlling for sex, age, race/ethnicity, marital status, education, MSA residence and number of chronic conditions. We did not control for insurance status because it is a major mediator in these associations.

Analyses were conducted in 2015. STATA statistical software (version 13.1; StataCorp, College Station, TX) was used in the 2-part modeling analysis. SAS and SAS-Callable SUDAAN (version 9.3; SAS Institute Inc, Cary, NC) were used in all other analyses. All estimates were weighted to account for the MEPS complex survey design and nonresponse.

## Results

Compared to the expansion group, the non-expansion group was significantly more likely to be female, black (25.6% vs. 16.8%), less educated, employed, living in non-MSA area (21.7% vs. 14.6%), uninsured (42.1% vs. 30.6%), and not currently enrolled in Medicaid (Table 1). No difference was observed between the two groups in terms of the number of chronic conditions. There are more obese people and smokers proportionally in the non-expansion group, although the difference was not statistically significant (Table 1).

**Table 1:** Demographic characteristics of low-income population aged 18–64, MEPS 2010–2012.

	Expansion		Non-expansion		
	(N = 9755; Weighted N = 22250481)		(N = 7457; Weighted N = 15865390)		P-value ^a^
Characteristic	Sample N	Weighted %	Sample N	Weighted %	
Gender					**0.0273**
Male	3963	45.2	2948	43.0	
Female	5792	54.8	4509	57.0	
Age					**0.049**
18–25	2374	25.7	1908	27.4	
26–29	982	10.0	737	9.6	
30–39	2286	20.8	1711	20.5	
40–49	1960	18.9	1441	19.2	
50–64	2153	24.6	1660	23.3	
Race/ethnicity					**< .0001**
Non-Hispanic white	2678	46.4	1959	46.8	
Non-Hispanic black	2117	16.8	2604	25.3	
Hispanic	4147	27.7	2606	23.3	
Other	813	9.0	288	4.6	
Marital status					0.1893
Not married	6679	70.0	4944	67.5	
Married	3076	30.0	2513	32.5	
Education					**0.0135**
Less than high school	3007	32.3	2369	32.5	
High school graduate	2586	33.2	2098	33.5	
Some college	1429	23.0	1142	25.4	
College graduate or more	615	11.5	371	8.6	
Employment status during the year					**0.0028**
Not employed	4770	48.0	3268	43.2	
Ever or currently employed	4969	52.0	4186	56.8	
Residence					**0.0218**
non-MSA	1045	14.6	1504	21.7	
MSA	8710	85.4	5953	78.3	
Health insurance					**< .0001**
Any private	1950	26.8	1729	30.2	
Public only	4466	42.6	2175	27.7	
Uninsured	3339	30.6	3553	42.1	
Ever have Medicaid during the year					**< .0001**
Yes	4484	42.4	2104	26.8	
No	5271	57.6	5353	73.2	
Number of chronic illnesses ^b^					0.6127
0	5246	50.3	3987	50.8	
1	1978	21.2	1478	20.2	
2+	2530	28.5	1991	29.0	
Weight status					0.1516
Underweight (BMI<18.5)	191	2.2	153	2.3	
Normal weight (18.5< = BMI<25)	2923	33.5	2155	31.8	
Overweight (25< = BMI<30)	3121	31.6	2268	30.3	
Obese (BMI> = 30)	3164	32.7	2649	35.6	
Currently smoke					0.124
Yes	2390	29.9	1978	32.8	
No	6608	70.1	4937	67.2	

MSA = Metropolitan statistical areas; BMI = Body mass index. For some factors, sum to less than total N because of missing values.

^a^ Wald Chi-square test; boldface indicates statistical significance (p<0.05).

^b^ Comorbid illnesses include: high blood pressure, stroke, heart disease, emphysema, high cholesterol, cancer, diabetes, arthritis and asthma.

Compared to the individuals in the expansion group, those in the non-expansion group were less likely to have a usual source of health care (64.5% vs. 56.5%, PR = 0.86, CI = 0.82–0.91) after controlling for demographic factors. They were also less likely to have dental checkups, routine physical checks, flu vaccinations and blood pressure checks as guidelines recommend (Table 2). No statistically significant difference was observed for cholesterol checks and cancer screening services (Table 2). Nevertheless, those in the non-expansion group were better off in one of the quality of care questions and mental health measures: among those who had medical appointments, a higher proportion of individuals in the non-expansion group reported always getting a medical appointment as soon as needed (Table 2); they were also less likely to report fair or poor mental health status compared to their expansion group counterparts (Table 2). In addition, no difference was observed between the expansion and non-expansion groups regarding attitudes about health (Table 2).

**Table 2:** Health-related outcomes by Medicaid expansion status among low income population aged 18–64 years, MEPS 2010–2012.

Health-related outcomes	Crude %		Adjusted Prevalence Ratio ^a^
	Expansion	Non-expansion	
***Perceived access to care***			
Usual source of health care	64.5 (62.7–66.3)	56.5 (54.0–59.0)	**0.86 (0.82–0.91)**
Unable to get or delayed any necessary medical care	10.1 (8.8–11.3)	11.0 (9.6–12.4)	1.13 (0.93–1.38)
Unable to get or delayed any necessary dental care	12.5 (10.9–14.2)	14.0 (12.1–15.9)	1.19 (0.98–1.43)
Unable to get or delayed any necessary prescription medication	7.7 (6.6–8.7)	7.7 (6.7–8.8)	1.00 (0.83–1.21)
***Realized access to preventive care***			
Dental checkup at least once a year	42.7 (41.0–44.5)	37.7 (35.2–40.3)	**0.86 (0.79–0.94)**
Routine check within past year	54.1 (52.3–55.9)	50.2 (46.7–53.7)	**0.89 (0.83–0.95)**
Flu vaccination within past year	27.7 (26.3–29.1)	24.8 (22.9–26.8)	**0.89 (0.81–0.98)**
Blood pressure check within past 2 years	82.2 (81.0–83.5)	80.6 (78.1–83.2)	**0.96 (0.94–0.99)**
Cholesterol check within past 2 years	54.8 (53.1–56.5)	53.6 (51.2–56.0)	0.99 (0.94–1.03)
Pap test within past 3 years ^b^	79.3 (77.5–81.2)	79.3 (77.2–81.4)	0.99 (0.95–1.03)
Mammogram within past 2 years ^b^	57.5 (54.8–60.3)	52.8 (48.8–56.8)	0.94 (0.85–1.04)
Blood stool test within past year ^b^	9.0 (7.5–10.5)	9.4 (7.2–11.6)	1.13 (0.85–1.51)
Sigmoidoscopy within past 5 years ^b^	4.7 (3.5–5.9)	4.0 (2.5–5.4)	1.02 0.66–1.57)
Colonoscopy within past 10 years ^b^	41.8 (38.4–45.1)	39.3 (35.8–42.8)	0.92 (0.80–1.05)
***Quality of Care (during past 12 months)***			
If had any condition needing care right away, always got care right away	45.9 (43.4–48.4)	46.6 (44.2–49.1)	0.97 (0.88–1.07)
If made any medical appointment, always got appointment as soon as needed	43.2 (41.0–45.3)	47.1 (44.7–49.5)	**1.08 (1.00–1.16)**
If any medical care was believed to be necessary, always easy getting care	48.5 (45.8–51.3)	52.2 (49.4–54.9)	1.03 (0.94–1.13)
***Attitudes about Health***			
Disagree strongly about "do not need health insurance"	58.3 (56.4–60.3)	57.3 (54.5–60.1)	0.97 (0.92–1.02)
Disagree strongly about "health insurance not worth cost"	34.7 (33.1–36.3)	34.1 (32.0–36.1)	0.95 (0.88–1.02)
Disagree strongly about "more likely to take risks"	34.0 (32.5–35.4)	33.5 (31.4–35.5)	0.95 (0.88–1.03)
Disagree strongly about "can overcome ills without med help"	43.9 (42.3–45.6)	42.6 (40.3–44.8)	0.95 (0.90–1.01)
***Health status***			
Fair/poor perceived health status	22.1 (20.6–23.5)	21.1 (19.6–22.6)	0.93 (0.85–1.03)
Fair/poor perceived mental health status	14.5 (13.5–15.6)	13.2 (11.7–14.8)	**0.86 (0.76–0.98)**
Worse Physicial Component Summary from SF-12v2	48.3 (46.6–49.9)	49.1 (47.0–51.3)	1.01 (0.95–1.06)
Worse Mental Component Summary from SF-12v2	45.9 (44.3–47.5)	43.1 (41.4–44.8)	**0.95 (0.90–1.00)**
Serious psychological distress (K6> = 13)	11.2 (10.1–12.2)	11.5 (10.3–12.7)	1.03 (0.91–1.17)
Depressive symptoms (PHQ2> = 3)	16.3 (15.1–17.5)	17.0 (15.5–18.4)	1.01 (0.91–1.11)

^a^ Models were adjusted for sex, age, race/ethnicity, marital status, education, metropolitan statistical areas residence and number of chronic conditions; prevalence ratio was for non-expansion vs. expansion group; boldface indicates statistical significance (p<0.05).

^b^ For Pap test, only women aged 21–64 without hysterectomy with a history of cervical cancer were included; for mammogram, only women aged 40–64 without a history of breast cancer were included; for colorectal cancer screening services, only adults aged 50–64 without a history of colorectal cancer were included.

On average, an individual in the non-expansion group had 1.16 (CI = 0.56–1.76) fewer office-based visits, 0.19 (CI = 0.09–0.28) fewer outpatient visits, 0.09 (CI = 0.05–0.41) fewer inpatient night stays, 0.08 (CI = 0.01–0.14) fewer dental visits, and 1.52 (CI = 0.05–2.98) fewer prescriptions annually than those in the expansion group (Table 3). The average annual medical expenditure for a low-income resident in the non-expansion group was $3421, compared to $4592 in the expansion group. The adjusted difference was $1410 (Table 3), which is 40% more than the per capita expenditure of the non-expansion group. When examined by service type, the expenditures were significantly higher in the expansion group for office-based, inpatient, and dental services, and prescriptions (Table 3). When examined by sources of payment, we found that on average private insurance spent similarly for a low-income individual in the two groups, while the government spent $1316 (CI $848-$1784) less for a person in the non-expansion group, and the out-of-pocket cost was significantly higher in the non-expansion group (Table 3).

**Table 3:** Number of care utilization and expenditures by Medicaid expansion status among low income population aged 18–64 years, MEPS 2010–2012.

Utilization and Expenditures	Crude mean ± Std		Adjusted Difference ^a^
	Expansion	Non-expansion	
***Annual care utilization by service type (Number)***			
Office-based visit	4.99 ± 0.23	3.92 ± 0.17	**1.16 (0.56–1.76)**
Outpatient visit	0.44 ± 0.04	0.28 ± 0.03	**0.19 (0.09–0.28)**
Emergency room visit	0.31 ± 0.01	0.31 ± 0.02	0.02 (-0.02–0.06)
Inpatient night stay	0.74 ± 0.07	0.53 ± 0.05	**0.09 (0.05–0.41)**
Dental visit	0.48 ± 0.02	0.38 ± 0.02	**0.08 (0.01–0.14)**
Prescription	13.06 ± 0.50	11.99 ± 0.74	**1.52 (0.05–2.98)**
***Annual medical expenditures ($)***			
Total	4592.19 ± 309.66	3420.54 ± 187.23	**1410.29 (781.63–2038.95)**
***By service type***			
Office-based	900.46 ± 49.04	634.10 ± 31.19	**300.27 (164.95–435.58)**
Outpatient	274.36 ± 30.44	244.41 ± 27.45	48.41 (-14.70–111.52)
Emergency room	233.52 ± 21.14	213.90 ± 14.16	21.50 (-20.42–63.41)
Inpatient	1475.19 ± 115.36	1095.53 ± 92.24	**138.88 (110.04–657.66)**
Dental care	135.80 ± 10.01	98.65 ± 9.10	**40.99 (14.00–67.98)**
Prescription	1307.46 ± 255.45	969.88 ± 82.96	**402.60 (47.81–757.39)**
***By source of payment***			
Out of pocket	376.46 ± 21.48	457.98 ± 33.06	**-97.17 (-158.75–-35.60)**
Private insurance + Tricare	747.90 ± 87.83	661.26 ± 78.16	190.82 (-39.70–421.35)
Government ^b^	2960.91 ± 258.62	1847.77 ± 152.19	**1315.76 (848.00–1783.52)**
Other sources ^b^	506.93 ± 55.94	453.56 ± 34.59	-63.43 (-177.26–50.40)

Std = standard deviation.

^a^ Two-part models were adjusted for sex, age, race/ethnicity, marital status, education, metropolitan statistical areas residence, and number of chronic conditions; difference was for expansion minus non-expansion group; boldface indicates statistical significance (p<0.05).

^b^ Government included Medicare, Medicaid, Veteran's Administration/CHAMPVA, and Workers' Compensation; Other sources include other federal, state, local, and unclassified sources.

## Discussion

This study presents a systematic comparison of the low-income adult populations by Medicaid state-level expansion status across multiple health-related outcomes, including access to care, preventive care receipt, care utilization and expenditures, quality of care, and certain self-reported health status. Our results showed that there were a higher proportion of low-income people in the nonexpanding states that did not have a usual source of care, did not meet guidelines for receipt of preventive services including dental checkups, routine physical checks, flu vaccinations and blood pressure checks. We also found that annual health care utilization and medical expenditures were on average significantly lower for low-income individuals in the non-expansion group; however, the out-of-pocket cost for the same group was significantly higher. The decision not to expand Medicaid may further increase these disparities between the two sets of states, especially when non-expanding states are disproportionately represented by disadvantaged segments of the population the blacks and rural residents. Furthermore, this may lead to unintended consequences in widening the black-white and rural-urban disparities in access to care and health outcomes nationwide.

The higher percentage of low income persons with a usual source of care and access to multiple preventive care services in Medicaid expanding states compared to those in the non-expanding states may in part reflect Medicaid prior expansion in certain ACA participating states. These differences between the two sets of states are likely to widen in the future in view of previous positive findings on expansion of medical care to the poor. For example, Sommers et al found that states that expanded adult Medicaid eligibility in the early 2000s experienced significantly reduced mortality as well as improved coverage, access to care and self-reported health [17]. Further, early provision of the ACA, including expansion of dependent coverage and elimination of cost sharing for preventive services, have shown to improve access to care [18, 19] and use of preventive services [20–22]. However, we found no difference in cancer screening services between Medicaid expansion and nonexpanding states. This may be in part due to the large and increasing number of public and private health targeted programs that provide breast and cervical screening for low income, uninsured and underinsured women, such as the CDC’s National Breast and Cervical Cancer Early detection Program (NBCCEDP) [23]. A recent study by Sabik et al. based on the 2012 Behavioral Risk Factor Surveillance System (BRFSS) data, however, found that women in the nonexpanding states had lower odds of receiving recommended mammograms or Pap tests [24]. The discrepancies in the findings between our study and Sabik et al. may be due to differences in study design (national-based vs. state-based), the mode of administration of the questionnaire (in-person vs. telephone interview, which is likely to overestimate screening utilization), and the choice of risk measurements (prevalence ratio vs. odds ratio, which tend to overestimate risk when the event is not rare [25]). Future studies should continue to monitor the disparities in cancer care and outcomes between the expanding and nonexpanding states.

We found that for most of the types of care, low-income people from nonexpanding states had a significantly lower utilization compared to those from the expanding states. The lower utilization of health care services may reflect a higher unmet need of health care among nonexpanding states’ residents. The total medical expenditure was accordingly lower in the nonexpanding states. When expenditures were examined by source of payment, the government in the nonexpanding states spent about $1300 less per person per year on their low-income residents than in the expanding states and private insurance spent similar amount, while the out-of-pocket expenditure per person per year was about $100 more for residents in nonexpanding states. Moving forward, with the expanding states continuing to increase their health care spending on low-income, uninsured residents through Medicaid expansion and the spending of nonexpanding states decreasing, the disparities in access to care, care utilization, and potential health care financial burdens that we observed in this baseline study seem likely to become wider.

Notwithstanding the nationally representative sample and the best available survey data on care utilization and medical expenditure, this study was limited on its health status component given the self-report nature of these outcomes. Particularly, we had limited variables on mental health despite it is an important topic as mental illness disproportionally affects low-income populations [26]. Future studies that utilize registry incidence, mortality data or clinical measures will help to investigate and monitor better the health status disparities by Medicaid expansion decision status in the low-income population. Another limitation is that we did not have homeless population in this household-based survey data, who will mostly become eligible under Medicaid expansion but actual enrollment will need targeted outreach and assistance [27].

In summary, this examination of many key baseline health characteristics immediately before the implementation of the ACA found that the low-income population in Medicaid nonexpanding states had worse access to care, less preventive care utilization, less medical expenditures and more out-of-pocket costs compared to those in expanding states. Unless the non-expanding states reverse their policies, their decisions may lead to widening of the disparities in access to care and outcomes in the low income population between the two sets of states, as well as the disparities between blacks and whites and between rural and urban residents nationwide.

## Supporting Information

S1 Appendix States’ population size, poverty rate and Medicaid expansion status. (DOCX)

## References

[1] Patient Protection and Affordable Care Act. Public Law No. 111–148, (2010).

[2] National Federation of Independent Business v. Sebelius: the Patient Protection and Affordable Care Act. Harvard law review. 2012;126(1):72–82. Epub 2012/11/01. . 25330555

[3] Statereform. Map: Where States Stand on Medicaid Expansion Decisions. Map updated November 2, 2015. [cited 2015 June 5]. Available from: https://www.statereforum.org/Medicaid-Expansion-Decisions-Map.

[4] Rasmussen PW, Collins SR, Doty MM, Beutel S. Health care coverage and access in the nation's four largest states. Results from the Commonwealth Fund Biennial Health Insurance Survey, 2014. Issue Brief (Commonw Fund). 2015;7:1–12. Epub 2015/04/22. . 25890978

[5] Agency for Healthcare Research and Quality. Medical Expenditure Panel Survey. [cited 2015 June 5]. Available from: http://meps.ahrq.gov/mepsweb/index.jsp.

[6] USPSTF A and B Recommendations. U.S. Preventive Services Task Force. October 2014. [cited 2015 April 28]. Available from: http://www.uspreventiveservicestaskforce.org/Page/Name/uspstf-a-and-b-recommendations/.

[7] Vaccine Recommendations of the ACIP. Advisory Committee for Immunization Practices (ACIP). Last updated March 30, 2015. [cited 2015 April 28]. Available from: http://www.cdc.gov/vaccines/hcp/acip-recs/index.html.

[8] Ware J Jr., Kosinski M, Keller SD. A 12-Item Short-Form Health Survey: construction of scales and preliminary tests of reliability and validity. Medical care. 1996;34(3):220–33. Epub 1996/03/01. . 8628042 10.1097/00005650-199603000-00003

[9] Ware JE Jr., Kosinski M, Turner-Bowker DM, Gandek B. User's Manual for the SF-12v2 Health Survey. Lincoln, RI: QualityMetric Incorporated; 2002.

[10] Kessler RC, Andrews G, Colpe LJ, Hiripi E, Mroczek DK, Normand SL, et al Short screening scales to monitor population prevalences and trends in non-specific psychological distress. Psychological medicine. 2002;32(6):959–76. Epub 2002/09/07. . 12214795 10.1017/s0033291702006074

[11] Kessler RC, Berglund PA, Glantz MD, Koretz DS, Merikangas KR, Walters EE, et al Estimating the prevalence and correlates of serious mental illness in community epidemiological surveys. In: Manderscheid RW, Henderson MJ, editors. Mental Health, United States, 2002 Rockville, Maryland: U.S. Department of Health and Human Services; 2004. p. 155–64.

[12] Kroenke K, Spitzer RL, Williams JB. The Patient Health Questionnaire-2: validity of a two-item depression screener. Medical care. 2003;41(11):1284–92. Epub 2003/10/30. . 14583691 10.1097/01.MLR.0000093487.78664.3C

[13] Agency for Healthcare Research and Quality. MEPS-HC-155 2012 full year consolidate data file documentation. [Internet]. Available from: http://meps.ahrq.gov/mepsweb/data_stats/download_data/pufs/h155/h155doc.shtml.

[14] Bieler GS, Brown GG, Williams RL, Brogan DJ. Estimating model-adjusted risks, risk differences, and risk ratios from complex survey data. American journal of epidemiology. 2010;171(5):618–23. Epub 2010/02/06. 10.1093/aje/kwp440 . 20133516

[15] Diehr P, Yanez D, Ash A, Hornbrook M, Lin DY. Methods for analyzing health care utilization and costs. Annual review of public health. 1999;20:125–44. Epub 1999/06/03. 10.1146/annurev.publhealth.20.1.125 . 10352853

[16] Honeycutt AA, Segel JE, Hoerger TJ, Finkelstein EA. Comparing cost-of-illness estimates from alternative approaches: an application to diabetes. Health services research. 2009;44(1):303–20. Epub 2009/01/17. 10.1111/j.1475-6773.2008.00909.x 19146569 PMC2669634

[17] Sommers BD, Baicker K, Epstein AM. Mortality and access to care among adults after state Medicaid expansions. N Engl J Med. 2012;367(11):1025–34. Epub 2012/07/27. 10.1056/NEJMsa1202099 . 22830435

[18] Barbaresco S, Courtemanche CJ, Qi Y. Impacts of the Affordable Care Act dependent coverage provision on health-related outcomes of young adults. J Health Econ. 2015;40:54–68. Epub 2015/01/17. 10.1016/j.jhealeco.2014.12.004 S0167-6296(14)00151-9 [pii]. . 25594956

[19] Sommers BD, Buchmueller T, Decker SL, Carey C, Kronick R. The Affordable Care Act has led to significant gains in health insurance and access to care for young adults. Health Aff (Millwood). 2013;32(1):165–74. Epub 2012/12/21. 10.1377/hlthaff.2012.0552 hlthaff.2012.0552 [pii]. . 23255048

[20] Fedewa SA, Goodman M, Flanders WD, Han X, Smith RA, E MW, et al Elimination of cost-sharing and receipt of screening for colorectal and breast cancer. Cancer. 2015 Epub 2015/06/05. 10.1002/cncr.29494 . 26042576

[21] Han X, Yabroff KR, Robbins AS, Zheng Z, Jemal A. Dependent coverage and use of preventive care under the Affordable Care Act. N Engl J Med. 2014;371(24):2341–2. Epub 2014/12/11. 10.1056/NEJMc1406586 25494285 PMC4283212

[22] Lau JS, Adams SH, Park MJ, Boscardin WJ, Irwin CE Jr. Improvement in preventive care of young adults after the affordable care act: the affordable care act is helping. JAMA Pediatr. 2014;168(12):1101–6. Epub 2014/10/28. 10.1001/jamapediatrics.2014.1691 1913624 [pii]. . 25347766

[23] National Breast and Cervical Cancer Early Detection Program (NBCCEDP) [cited 2015 June 1]. Available from: http://www.cdc.gov/cancer/nbccedp/data/summaries/national_aggregate.htm.

[24] Sabik LM, Tarazi WW, Bradley CJ. State Medicaid expansion decisions and disparities in women's cancer screening. Am J Prev Med. 2015;48(1):98–103. Epub 2014/12/03. 10.1016/j.amepre.2014.08.015 S0749-3797(14)00482-6 [pii]. 25441234 PMC4274203

[25] Davies HT, Crombie IK, Tavakoli M. When can odds ratios mislead? BMJ. 1998;316(7136):989–91. Epub 1998/04/29. PubMed Central PMCID: PMC1112884. 9550961 10.1136/bmj.316.7136.989 PMC1112884

[26] Tsai J, Pilver CE, Hoff RA. Potential mental health needs of US adult residents under different provisions of the Affordable Care Act. J Clin Psychiatry. 2014;75(12):1402–10. Epub 2014/10/02. 10.4088/JCP.13m08885 . 25271598

[27] Tsai J, Rosenheck RA, Culhane DP, Artiga S. Medicaid expansion: chronically homeless adults will need targeted enrollment and access to a broad range of services. Health Aff (Millwood). 2013;32(9):1552–9. Epub 2013/09/11. 10.1377/hlthaff.2013.0228 32/9/1552 [pii]. . 24019359

