# Supplementary material for: Health-Related Outcomes among the Poor: Medicaid Expansion vs. Non-Expansion States
Source: PLoS One. 2015 Dec 31;10(12):e0144429. doi: 10.1371/journal.pone.0144429 (PMC4700996; doi:10.1371/journal.pone.0144429)
Supplement: S1 Appendix — (DOCX) [file pone.0144429.s001.docx]

S1 Appendix. States' Population Size, Poverty Rate and Medicaid Expansion status

|  |  |  |  |
| --- | --- | --- | --- |
| **State** | **Population** | **Percent below poverty** | **Medicaid expansion status** |
| Alabama | 4,849,377 | 19.3 | N |
| Alaska | 736,732 | 11.2 | N |
| Arizona | 6,731,484 | 18.2 | Y |
| Arkansas | 2,966,369 | 18.9 | Y |
| California | 38,802,500 | 16.4 | Y |
| Colorado | 5,355,866 | 12 | Y |
| Connecticut | 3,596,677 | 10.8 | Y |
| Delaware | 935,614 | 12.5 | Y |
| District of Columbia | 658,893 | 17.7 | Y |
| Florida | 19,893,297 | 16.5 | N |
| Georgia | 10,097,343 | 18.3 | N |
| Hawaii | 1,419,561 | 11.4 | Y |
| Idaho | 1,634,464 | 14.8 | N |
| Illinois | 12,880,580 | 14.4 | Y |
| Indiana | 6,596,855 | 15.2 | Y |
| Iowa | 3,107,126 | 12.2 | Y |
| Kansas | 2,904,021 | 13.6 | N |
| Kentucky | 4,413,457 | 19.1 | Y |
| Louisiana | 4,649,676 | 19.8 | N |
| Maine | 1,330,089 | 14.1 | N |
| Maryland | 5,976,407 | 10.1 | Y |
| Massachusetts | 6,745,408 | 11.6 | Y |
| Michigan | 9,909,877 | 16.2 | Y |
| Minnesota | 5,457,173 | 11.5 | Y |
| Mississippi | 2,994,079 | 21.5 | N |
| Missouri | 6,063,589 | 15.5 | N |
| Montana | 1,023,579 | 15.4 | N |
| Nebraska | 1,881,503 | 12.4 | N |
| Nevada | 2,839,099 | 15.2 | Y |
| New Hampshire | 1,326,813 | 9.2 | Y |
| New Jersey | 8,938,175 | 11.1 | Y |
| New Mexico | 2,085,572 | 21.3 | Y |
| New York | 19,746,227 | 15.9 | Y |
| North Carolina | 9,943,964 | 17.2 | N |
| North Dakota | 739,482 | 11.5 | Y |
| Ohio | 11,594,163 | 15.8 | Y |
| Oklahoma | 3,878,051 | 16.6 | N |
| Oregon | 3,970,239 | 16.6 | Y |
| Pennsylvania | 12,787,209 | 13.6 | Y |
| Rhode Island | 1,055,173 | 14.3 | Y |
| South Carolina | 4,832,482 | 18 | N |
| South Dakota | 853,175 | 14.2 | N |
| Tennessee | 6,549,352 | 18.3 | N |
| Texas | 26,956,958 | 17.2 | N |
| Utah | 2,942,902 | 11.7 | N |
| Vermont | 626,562 | 12.2 | Y |
| Virginia | 8,326,289 | 11.8 | N |
| Washington | 7,061,530 | 13.2 | Y |
| West Virginia | 1,850,326 | 18.3 | Y |
| Wisconsin | 5,757,564 | 13.2 | N |
| Wyoming | 584,153 | 11.2 | N |
|  |  |  |  |

Population size and poverty rate were estimated through 2014 American community Survey by U.S. Census Bureau. See details <http://factfinder.census.gov/faces/nav/jsf/pages/index.xhtml>

Medicaid expansion status was as of February 2015, the time of analysis. Montana and Alaska moved to participate in Medicaid expansion in April and July 2015 respectively.
